# Supplementary material for: Dynalign II: common secondary structure prediction for RNA homologs with domain insertions
Source: Nucleic Acids Res. 2014 Nov 21;42(22):13939–48. doi: 10.1093/nar/gku1172 (PMC4267632; doi:10.1093/nar/gku1172)
Supplement: SUPPLEMENTARY DATA [file supp_gku1172_nar-02021-z-2014-File012.zip › manual/Text/EnergyPlot.html]

RNAstructure Command Line Help: EnergyPlot


|  |  |  |
| --- | --- | --- |
|  | RNAstructure Command Line Help EnergyPlot | - Contents - Index |
| EnergyPlot is used to create a folding free energy plot generated after determining the structure of a strand or strands of nucleic acids. USAGE: EnergyPlot <save file> <ps file> [options] Required parameters: <save file> \| The name of a binary folding save file containing free energy data from a previous folding calculation. These folding free energy save files usually end in ".sav".; <output file> \| The name of a file to which output will be written. Options which don't require added values: -h, -H, --help \| Display the usage details message.; --svg \| Specify that the output file should be an SVG image file, rather than a Postscript image file.; -t, -T, --text \| Write dot plot as a text file, rather than a Postscript image file. Options which require added values: -e, -E, --entries \| Specify the number of categories in the plot legend. Default is 5 entries.; -max, -MAX, --maximum \| Specify the maximum value in the plot in kcal/mol. Default is the largest valid value in the plot.; -min, -MIN, --minimum \| Specify the minimum value in the plot in kcal/mol. Default is the smallest valid value in the plot. References: 1. Reuter, J.S. and Mathews, D.H. "RNAstructure: software for RNA secondary structure prediction and analysis." *BMC Bioinformatics*, 11:129. (2010). 2. Zuker, M. "On finding all suboptimal foldings of an RNA molecule." *Science*, 244:48-52. (1989). | | |
| Visit The Mathews Lab RNAstructure Page for updates and latest information. | | |
